# Supplementary material for: Angelica sinensis polysaccharide as potential protectants against recurrent spontaneous abortion: focus on autophagy regulation
Source: Front Med (Lausanne). 2025 Jan 15;12:1522503. doi: 10.3389/fmed.2025.1522503 (PMC11774876; doi:10.3389/fmed.2025.1522503)
Supplement: Supplementary material S1 — The main instruments used during the LC-MS process, along with their models/specifications and manufacturers. [file Supplementary_file_1.zip › Metabolomics sequencing data FC1.2/8.代谢通路分析/Exposure_Ctrl/all/KEGG/mmu04072.html]

KEGG PATHWAY: Phospholipase D signaling pathway - Mus musculus (house mouse)


# Phospholipase D signaling pathway - Mus musculus (house mouse)


[
Pathway menu
| Organism menu
| Pathway entry
| Show description
| Download
| Help
]

Phospholipase D (PLD) is an essential enzyme responsible for the production of the lipid second messenger phosphatidic acid (PA), which is involved in fundamental cellular processes, including membrane trafficking, actin cytoskeleton remodeling, cell proliferation and cell survival. PLD activity can be stimulated by a large number of cell surface receptors and is elaborately regulated by intracellular factors, including protein kinase C isoforms, small GTPases of the ARF, Rho and Ras families and the phosphoinositide, phosphatidylinositol 4,5-bisphosphate (PIP2). The PLD-produced PA activates signaling proteins and acts as a node within the membrane to which signaling proteins translocate. Several signaling proteins, including Raf-1 and mTOR, directly bind PA to mediate translocation or activation, respectively.


##### Option

Scale:


100%

Image resolution:


 High

##### Search

##### ID search

##### Color


KGML

Image (png) file 1x
